# Supplementary material for: Evidence for Toxic Advanced Glycation End-Products Generated in the Normal Rat Liver
Source: Nutrients. 2019 Jul 16;11(7):1612. doi: 10.3390/nu11071612 (PMC6683103; doi:10.3390/nu11071612)
Supplement: Supplementary file 1 [file nutrients-11-01612-s001.pdf]

Supplementary

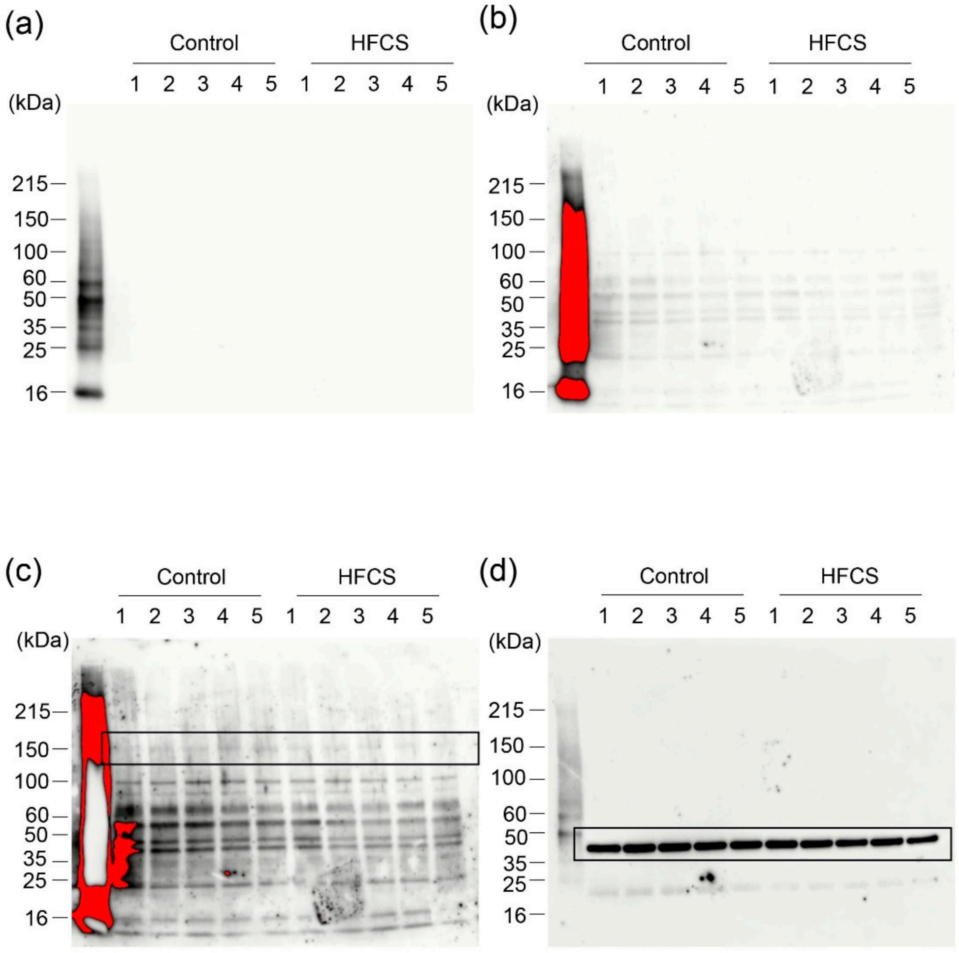

Figure S1. Full-length blots of Figure 4a. Tissue lysates were loaded on a 4–15% gradient sodium dodecyl sulphate-polyacrylamide (SDS-PAGE) gel. (**a**) The type IV collagen band was detected on the polyvinylidene difluoride (PVDF) membrane with high sensitivity and an exposure time of 1.0 s. (**b**) The type IV collagen band was detected on the PVDF membrane with high sensitivity and an exposure time of 10 s. (**c**) The type IV collagen band was detected on the PVDF membrane with high sensitivity and an exposure time of 60 s. The open box replaces the type IV collagen band indicated in Figure 4a. (**d**) The β-actin band was detected on the PVDF membrane. The open box replaces the β-actin band indicated in Figure 4a.

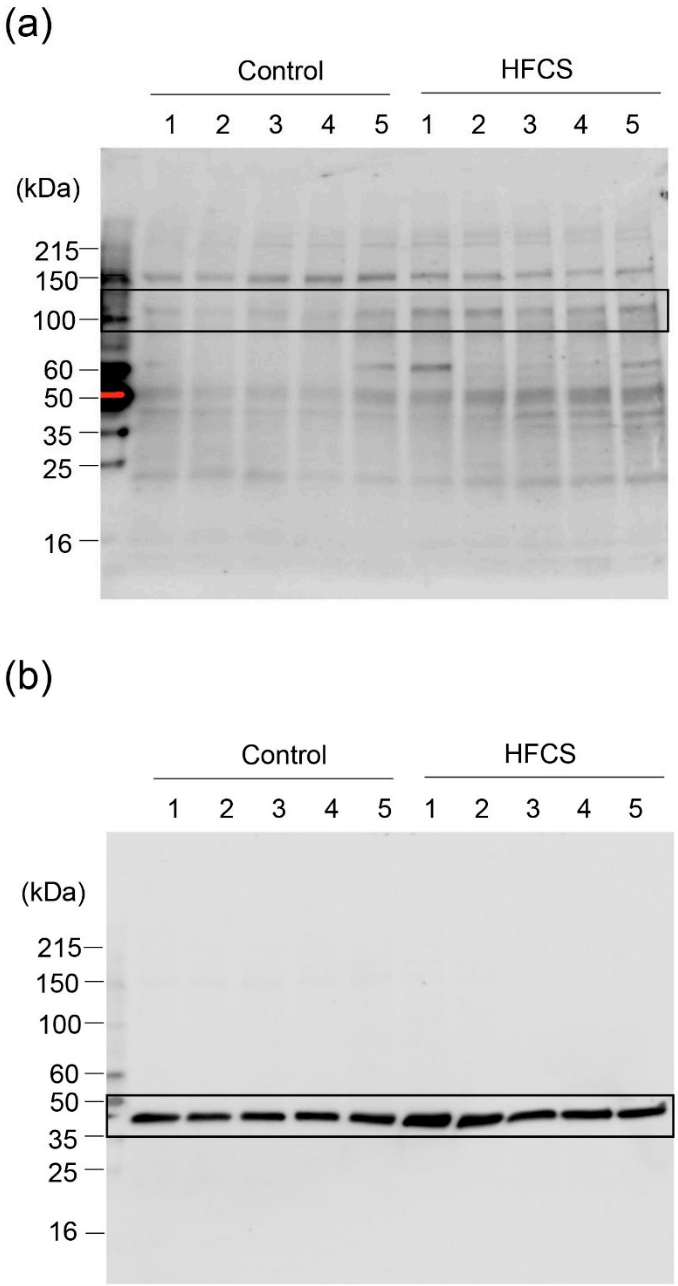

Figure S2. Full-length blots of Figure 4b. Tissue lysates were loaded on a 4–15% gradient SDS-PAGE gel. (**a**) The SIRT1 band was detected on the polyvinylidene difluoride (PVDF) membrane. The open box replaces the SIRT1 band indicated in Figure 4b. (**b**) The β-actin band was detected on the PVDF membrane. The open box replaces the β-actin band indicated in Figure 4b.

(a)

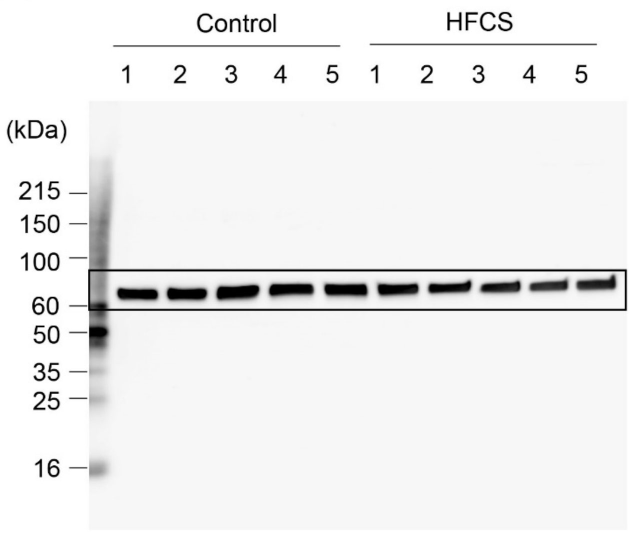

(b)

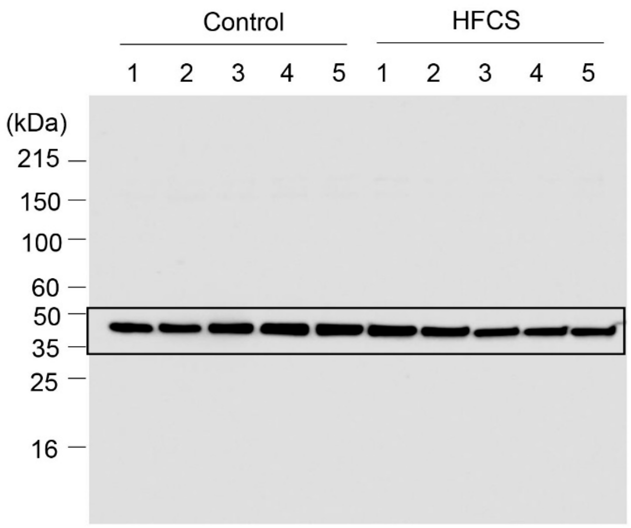

Figure S3. Full-length blots of Figure 4c. Tissue lysates were loaded on a 4–15% gradient SDS-PAGE gel. (**a**) The HSP70 band was detected on the PVDF membrane. The open box replaces the HSP70 band indicated in Figure 4c. (**b**) The β-actin band was detected on the PVDF membrane. The open box replaces the β-actin band indicated in Figure 4c.

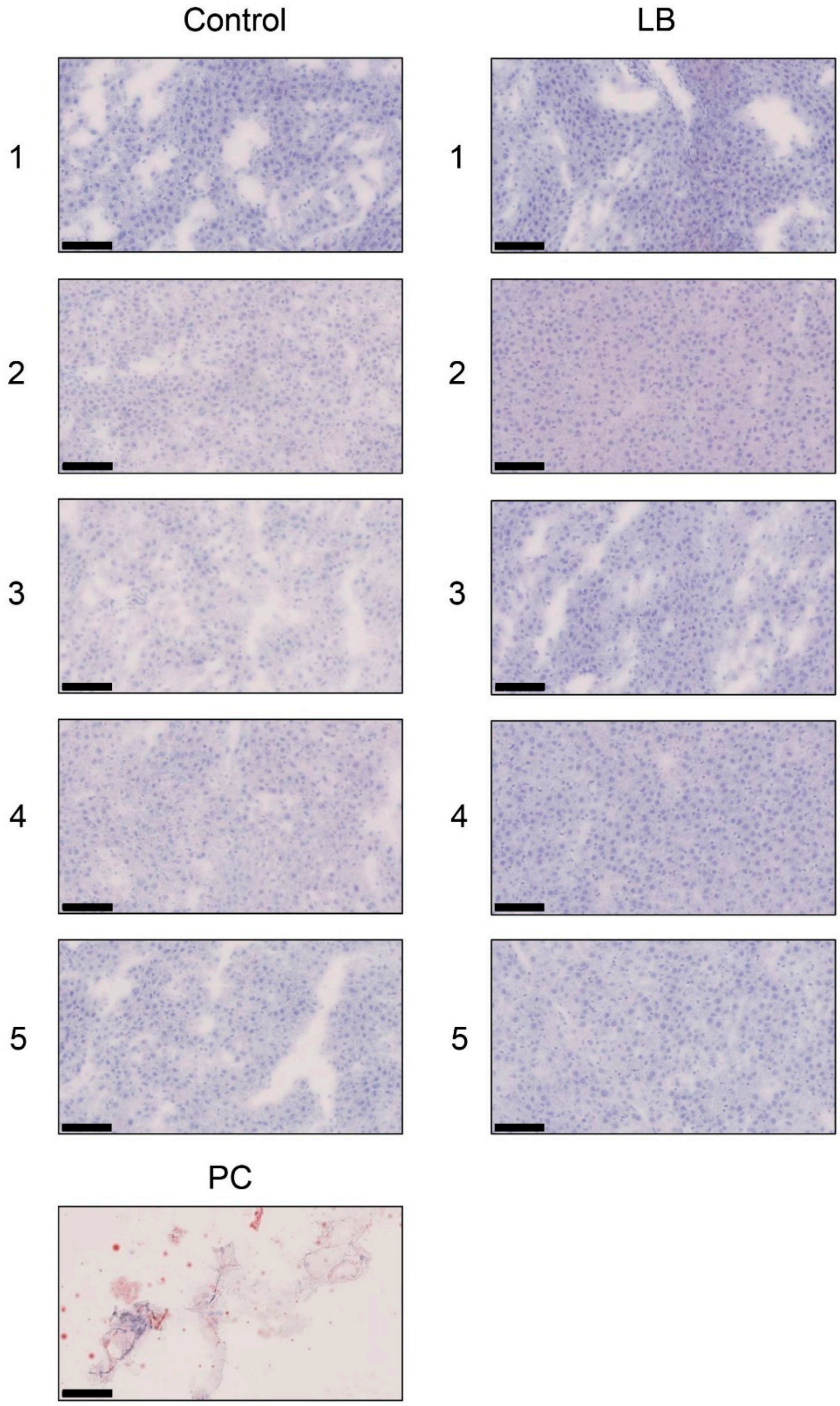

Figure S4. Oil Red O staining of the liver. The numbers 1–5 indicate each rat in the control group (n = 5) or *Lactobacillus* beverage group (n = 5). LB; *Lactobacillus* beverage group. PC; Positive control. Adipose tissue of rat. Oil Red-positive areas stained red in cells. The scale bar represents 100 μm.

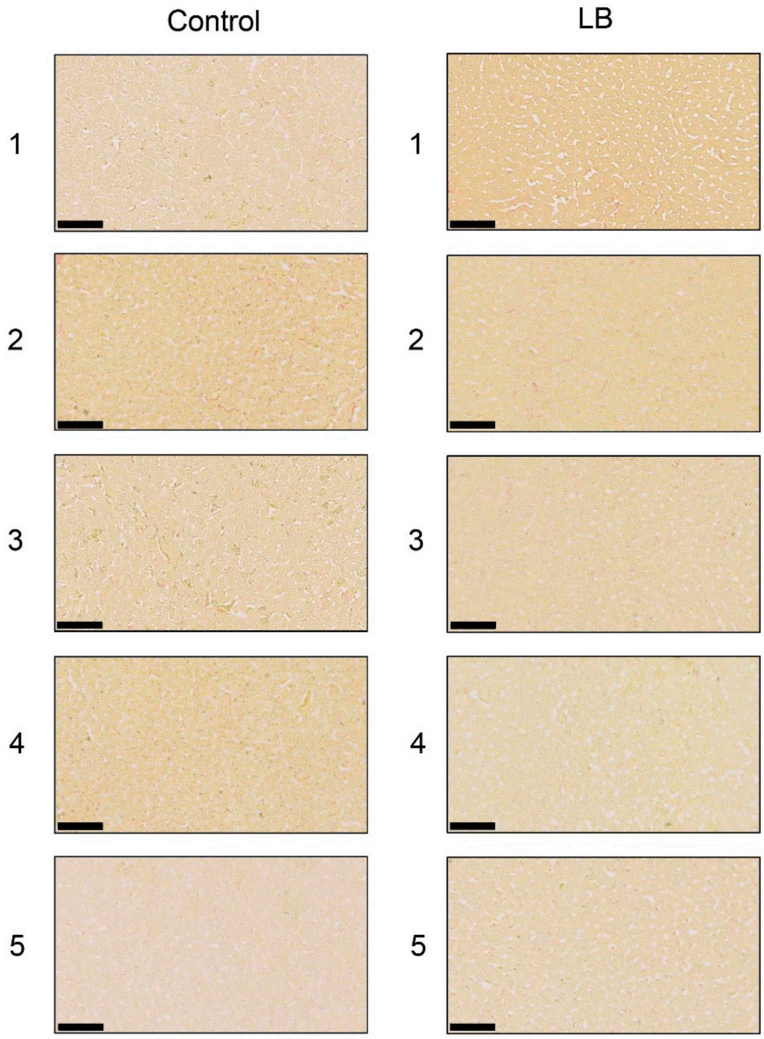

Figure S5. Sirius Red staining of the liver. The numbers 1–5 indicate each rat in the control group (n = 5) or *Lactobacillus* beverage group (n = 5). LB; *Lactobacillus* beverage group. Type I or III collagen stained red. Muscle fibers and cytoplasm stained yellow. The scale bar represents 100 μm.

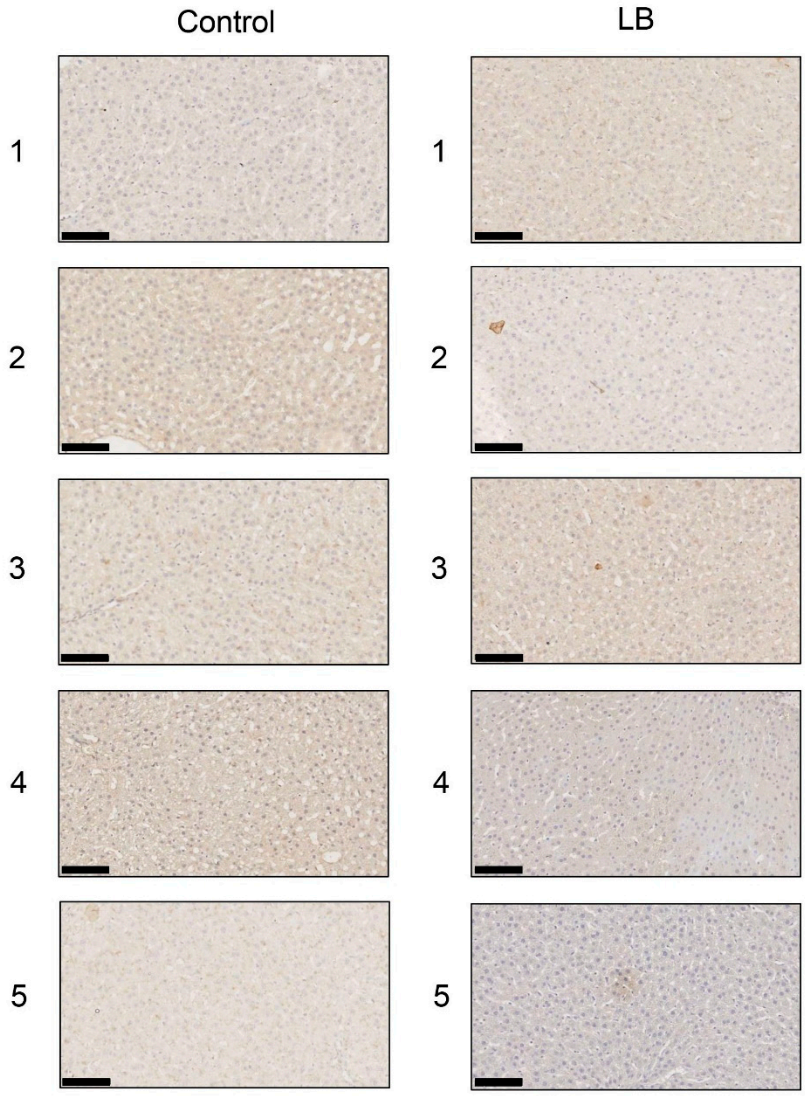

Figure S6. Immunostaining of intracellular TAGE in the liver. The numbers 1–5 indicate each rat in the control group (n = 5) or *Lactobacillus* beverage group (n = 5). LB; *Lactobacillus* beverage group. TAGE-positive areas stained brown in cells. The scale bar represents 100 μm.
